# The Career of the Medical Student

**Published:** 1896-09-05

**Authors:** 


### The Career of the Medical Student.

THE CHOICE OF A QUALIFICATION.

When a man has decided to enter upon the medical profession it becomes a matter of considerable importance that he should make up his mind from the beginning what sort of a position it is at which he aims. Some men's ambitions are limited to small things; a simple life with but few pleasures, and those of *bourgeois* sort, is sufficient for them. They have a mind above the shop, but not much above it—a side entrance will satisfy them; they aspire to a carriage, but a gig will do; they do not like a red bottle in the window, but they temporise by putting up a red lamp. For the satisfaction of such ambitions it seems a pity to indulge in university degrees or expensive diplomas. These people are, in fact, the representatives in modern life of the apothecaries of old; they fill a useful and a perfectly honourable position in the social system, and the licence of the Society of Apothecaries is their proper qualification. There are others who aim at good-class family practice, whose fate it will be to become leading men in small country towns, mixing on terms of equality with all the best people round about. To such men the power of becoming F.R.C.S. or M.R.C.P.—diplomas the value and meaning of which are ill-understood by the people—is as nothing compared with the possession of an M.D. degree, and the undoubted right to be called "Doctor," together with that tone of thought and mode of looking at things which are to be gained by passing one's student days at a university.

There are others again who aim at higher things, who wish to become consultants, to get on the staff at great hospitals, and to become famous as teachers and leaders in their profession. To all such it should be known at once that such positions in London are open solely to those who are members of the Royal College of Physicians of London or Fellows of the Royal College of Surgeons of England. Without one or other of these diplomas, according as medicine or surgery is to be taken up, no one can get on to the staff of any of the London hospitals; and, therefore, if he wishes for such a position, whatever other degrees he may take, he must arrange that his curriclum shall fall in with the acquisition of these essential qualifications.

At the same time it must be remembered that a consulting physician without a university degree would be an anomalous creature. Custom goes for much and is hard to kick against, and the custom is for physicians to possess a degree in medicine.

If, then, a man wishes to be able to fill the highest positions in the profession, supposing the fates should smile upon him, he must so arrange his course of study as to become F.R.C.S., Eng., if surgery is to be his vocation, and, if medicine, then M.R.C.P., Lond., and to obtain, in addition, a university degree in medicine.

It is to be hoped that when the Bill is passed, which is now before Parliament, for the creation of a teaching university for London, it will be easier than it now is for the London student to work at the same time for the degree and the diploma. At present, in view of the fact that the London conjoint diploma can be obtained after studying at any medical school in either England, Scotland, ro Ireland, the choice of place of study really depends upon where one can best obtain the university degree.

In many provincial towns medical schools have been established, which compare favourably with some of those in the metropolis. But it is quite certain that a man educated entirely in the provinces would find it difficult to obtain that footing on the lower rungs of the ladder which is necessary for those who aim at hospital appointments in London. We would therefore advise the student who aims at the higher professional life in London to go through his purely medical studies at a London hospital.

The question then is, Where should he get his degree?

To those who are able to pass the examinations of the University of London no better degree could be offered. It has, however, the reputation of being difficult to obtain.

The next alternative is to go to Cambridge, and after either obtaining the ordinary B.A., or preferably after going out in the Natural Science Tripos, going to London, and finishing the medical curriculum at one of the London hospitals.

By matriculating at Durham, or at the Victoria University, or at one of the Scotch Universities, and spending a portion of the time at one or other of these places (one year in the case of Durham, and two years in either of the others), the same end may be gained with less loss of time; that is, the student can then come to London for the rest of his curriculum, and can take up his degree without further residence at his university.

For those who can afford it we certainly should recommend Cambridge; while for those who can do it there are undoubted advantages in the London degree, partly in the degree itself, and partly from the fact that when the whole of the curriculum is gone through at one school, if the student does well, he has a greater chance of becoming known to those in authority.

With the object, then, of being prepared to take the highest positions in the profession the young man who intends to enter the medical profession will do well to matriculate either at London or Cambridge, and while following the prescribed course for his degree to pass at the proper stages the examinations for the conjoint diploma of the Royal Colleges of Physicians and Surgeons. This is the first step to the higher qualifications of both these colleges.

For those whose aim is general practice the desirability of possessing an M.D. is such that it merely becomes a question at which university it should be obtained. In deciding this the very great attractions of Edinburgh as a place for medical study must be remembered, while the cheapness of living at Aberdeen is a matter which some may find worthy of consideration: at the same time, there are many social advantages, perhaps more in the case of private practice than in consulting work, where one always in a manner speaks *ex cathedra*, in having passed one's student life at one of the older English universities.

Lastly, for those whose sole ambition is to earn a living in low-class general practice in a large town the cheapest and most easily obtained qualification would seem the most suitable, although not a few people tell us that even in this style of practice a university degree has a certain commercial value. We would repeat, however, that for the man who enters with no special aim, and, as is so often the case, without the remotest idea what are his powers, or wherein they mostly lie, the most desirable course is to arrange his studies in such a fashion that he shall be able to take advantage of whatever opportunities fortune may throw in his way, and that can best be done by passing the conjoint examination of the London colleges, and taking a degree.

